# Inhibition of HMOX1 alleviates diabetic cardiomyopathy by targeting ferroptosis: Inhibition of HMOX1 alleviates diabetic cardiomyopathy

**DOI:** 10.3724/abbs.2024232

**Published:** 2025-04-16

**Authors:** Huiping Yang, Gongyi Xiao, Dinghui Wang, Tianhua Xiong, Jing Wang, Xiaodong Jing, Bingquan Xiong, Junmei Xie, Bin Liu, Qiang She

**Affiliations:** 1 Department of Cardiology the Second Affiliated Hospital of Chongqing Medical University Chongqing 400010 China; 2 Department of Orthopedic Surgery the Second Affiliated Hospital of Chongqing Medical University Chongqing 400010 China; 3 Department of Orthopedic Surgery Chonggang General Hospital Chongqing 400000 China

**Keywords:** diabetic cardiomyopathy, ferroptosis, H9C2, HMOX1, GPX4, lipid peroxidation

## Abstract

Diabetic cardiomyopathy (DCM) is an important complication of chronic diabetes mellitus. However, its pathologic process and pathogenesis have not been fully elucidated. This study aims to investigate the role of ferroptosis in DCM and clarify the effect of heme oxygenase-1 (HMOX1) on DCM by targeting ferroptosis. *In vivo*, an animal model of DCM is established by subjecting mice to a high-fat diet (HFD) combined with low-dose streptozotocin (STZ) injection. We induce an *in vitro* DCM model by exposing H9C2 cells to high glucose and palmitic acid. Transcriptome sequencing reveals that the differentially expressed genes (DEGs) are enriched primarily in fatty acid metabolism and mitochondrial fatty acid β-oxidation, which are closely related to ferroptosis. The experimental results show that the diabetic microenvironment induces ferroptosis both *in vivo* and *in vitro*. Western blot analysis reveals the decreased expressions of the antioxidant proteins GPX4, SLC7A11 and ferritin in the DCM group. However, qPCR demonstrates the elevated expressions of the ferroptosis markers *PTGS2* and *ACSL4*. Biochemical indicators further support the occurrence of ferroptosis, with increased levels of malondialdehyde (MDA) and lactate dehydrogenase (LDH), along with decreased level of glutathione (GSH). *In vitro*, intervention with high glucose and palmitic acid in H9C2 cells results in ferroptosis, which is reversed by ferrostatin-1 (Fer-1). Results show the elevated expression of HMOX1 in DCM. Moreover, knockdown of *HMOX1* ameliorates ferroptosis, thereby alleviating diabetic cardiomyopathy by reducing cardiac fibrosis and improving cardiac function. Our study elucidates the role of HMXO1 in DCM pathogenesis and provides a potential therapeutic strategy for clinical treatment.

## Introduction

Over the past few decades, diabetes mellitus (DM) has emerged as a global epidemic due to lifestyle changes, technological advancements and social development [1]. DM is recognized as an independent risk factor for cardiovascular diseases across different ethnic groups and sexes [2]. Type 2 diabetes is characterized by metabolic disorders, such as obesity and insulin resistance, leading to abnormal glucose and dyslipidemia in various organs, including the heart [3]. Diabetic cardiomyopathy (DCM) is a primary myocardial injury induced by DM in the absence of coronary artery disease, hypertension, dyslipidemia and valvular heart disease [4]. DCM has a complex pathogenesis, initially manifesting as diastolic relaxation abnormalities and progressing to clinical heart failure [5]. However, the underlying pathophysiological mechanisms of DCM are not yet fully understood. Currently, DCM treatment focuses on controlling blood glucose, blood pressure, and lipid levels and improving lifestyle. Specific targeted treatment methods are not yet available, and the incidence and mortality rates of heart failure remain high.

Ferroptosis, which was first defined by Dr. Brent R Stockwell in 2012 [6], represents a unique form of cell death driven by iron-dependent phospholipid peroxidation [7]. It is characterized by the inactivation of cellular glutathione (GSH), depletion of glutathione peroxidase 4 (GPX4), and accumulation of toxic lipid-reactive oxygen species (ROS) [8]. Ferroptosis has been implicated in various types of pathological cell death associated with carcinogenesis, stroke, degenerative diseases, ischaemia‒reperfusion injury, and kidney degeneration [9]. In recent years, the role of ferroptosis in cardiovascular diseases (CVDs) has been highlighted, suggesting its potential as a therapeutic target for CVD [10]. Emerging evidence indicates the involvement of ferroptosis in diabetic complications due to cellular metabolic disorders [11]. However, the association between ferroptosis and DCM has not been extensively explored [12].

In the present study, we successfully established a DCM mouse model and confirmed the crucial role of ferroptosis in the diabetic microenvironment both *in vivo* and *in vitro*. Mechanistically, the upregulation of heme oxygenase-1 (HMOX1) leads to heme degradation and intracellular iron overload. Inhibition of HMOX1 rescues H9C2 cell death and alleviates DCM by targeting ferroptosis. Our findings provide potential therapeutic targets for the prevention and treatment of DCM.

## Materials and Methods

### Animals and ethics statement

All the animal experiments were conducted in strict compliance with the ethical guidelines outlined in the National Institutes of Health Guide for the Care and Use of Laboratory Animals and the ARRIVE (Animal Research: Reporting In Vivo Experiments). The experimental protocols were approved by the Animal Ethics Committee of Chongqing Medical University (Approval number: IACUC-CQMU-2023-0257). Three-week-old male C57BL/6J mice were procured from Chongqing Medical University Animal Research Center and housed in pathogen-free facilities at 24 ± 2°C with 55% ± 5% humidity and a 12/12 light/dark cycle. All the mice were provided with water *ad libitum*.

### Construction of the DCM mouse model and drug experiments

After a one-week period of acclimatization, the mice were randomly divided into two groups. One group received a high-fat diet (HFD, 60% kcal% fat), while the other group was fed with a normal chow diet. At the age of 12 weeks, the HFD-fed mice were intraperitoneally injected with a low dose of streptozotocin (STZ, 40 mg/kg, dissolved in 0.1 M citrate acid buffer; #60256ES80; Yeasen, Shanghai, China) for 3 consecutive days. The mice fed with a normal diet received injections of only citrate acid buffer. Seven days after STZ administration, fasting blood glucose (FBG) levels were measured. Mice with a blood glucose concentration  ≥ 11.1 mM were included in the DCM group. The control + Fer-1 and DCM + Fer-1 groups subsequently received intraperitoneal injections of Fer-1 (10 mg/kg, dissolved in DMSO) every other day for two months. The control + shHMOX1 and DCM + shHMOX1 groups were administered AAV9 carrying the HMOX1 interference sequence via tail vein injection. The AAV9 interference sequence was 5′-AGCCACACAGCACTATGTAAA-3′. All the mice were continually fed with a HFD or a normal diet until they were sacrificed at the age of 25 weeks for further analysis.

### IPGTT and IPITT

In the final week of the treatment period, following an overnight fasting for 12 h, both an intraperitoneal glucose tolerance test (IPGTT) and an intraperitoneal insulin tolerance test (IPITT) were conducted on all the mice. For the IPGTT, each mouse received an intraperitoneal injection of 1 mg/g glucose. Blood glucose levels were measured at 0, 15, 30, 60 and 120 min after injection. Similarly, for the IPITT, the mice were administered with 1 U/kg of insulin through an intraperitoneal injection. Blood glucose levels were then measured at 0, 15, 30, 60, and 120 min after insulin injection.

### Echocardiography

The mice were carefully anesthetized with 0.3% pentobarbital sodium. Transthoracic echocardiography was then performed via the small animal ultrasound imaging system (#VINNO 6 LAB, Nanjing, China). The echocardiograms were conducted by highly skilled and experienced echocardiographers who were unaware of the grouping of the mice, ensuring unbiased assessment. The left ventricular ejection fraction (LVEF), fractional shortening (FS), systolic left ventricle posterior wall thickness (LVPWs), diastolic left ventricle posterior wall thickness (LVPWd *)*, left ventricular internal diameter in systole (LVIDs) and left ventricular internal diameter in diastole (LVIDd) were measured by M-mode echocardiography in the left ventricular long-axis view.

### RNA-sequencing analysis

RNA-sequencing analysis was conducted on heart tissues from both the diabetic and control groups, with each group consisting of 3 biological replicates to ensure the robustness of the results. Total RNA was extracted using Trizol reagent (Invitrogen, Carlsbad, USA). RNA purification, reverse transcription, library construction and sequencing were then carried out using the Illumina NovaSeq 6000 system at Majorbio Biopharm Biotechnology Co., Ltd. (Shanghai, China). Genes with *P* values less than 0.05 and log _2_FC > 1 were considered as differentially expressed genes (DEGs). To gain further insight into the functional implications of the DEGs, Gene Ontology (GO) analysis and Reactome enrichment analysis were performed.

### Cell culture and treatment

H9C2 cells (#TCR-C607) were purchased from HyCyte (Nanjing, China). The cells were cultured in DMEM supplemented with 10% fetal bovine serum (FBS; #10099-141; Gibco, Carlsbad, USA), 1% L-glutamine (#C0212; Beyotime, Shanghai, China) and 1% penicillin-streptomycin (#HY-K1006; MCE, Shanghai, China) at 37°C in 5% CO _2_. The cells were treated with 25.5 mM glucose (control group), 25.5 mM glucose/34.5 mM mannitol/20% BSA (hypertonic solution and solvent control group), 60 mM glucose/0.1 mM palmitic acid (GP group) or GP/10 μM ferrostatin (Fer-1, dissolved in DMSO, pretreated for 2 h; #GC10380; GLPBIO, Montclair, USA). The palmitate was dissolved in 0.1 M NaOH solution and 20% BSA to make a 100 mM stock solution. The mixture was shaken in a 55°C water bath for 30 min and then filtered through a 0.22-μm sterile filter.

### Cell viability

Cell viability was evaluated by CCK-8 assay. In brief, H9C2 cells were cultivated at a density of 3 × 10 ^3^ cells/well in a 96-well plate and treated for 24, 48, or 72 h. After that, the culture medium was replaced by fresh working solution containing 10% CCK-8 reagent (#GK10001; GLPBIO). The cells were subsequently incubated at 37°C for 2 h. The absorbance at 450 nm was subsequently measured via a microplate reader.

### Western blot analysis

To analyze protein expression, heart tissues and H9C2 cells were lysed with RIPA lysis buffer (#AR0102; BOSTER, Wuhan, China), followed by centrifugation at 13,000 *g* for 15 min at 4°C. The resulting supernatant was mixed with 5× SDS-PAGE loading buffer (#AR1112; BOSTER) and boiled in a water bath for 10 min. Each sample, which contained 20 μg of total protein per sample, was then separated via 10% Tris-glycine SDS-PAGE and transferred to a 0.45-μm PVDF membrane (#IPVH00010; Millipore, Billerica, USA). Next, the membranes were blocked with 5% skim milk at room temperature for 2 h and then incubated with primary antibodies overnight at 4°C. After that, the membranes were rinsed and further incubated with horseradish peroxidase-conjugated secondary antibody for 2 h at room temperature. The membranes were subsequently washed with Tris-buffered saline containing 1‰ Tween 20. Finally, the protein signals were visualized via a ChemiDoc imaging system (Bio-Rad, Hercules, USA). The protein expression levels were normalized to those of β-actin. The following antibodies were used: HMOX1 (dilution 1:2000, #ab189491; Abcam, Cambridge, UK), GPX4 (dilution 1:2000, #ab125066; Abcam), solute carrier family 7 member 11 (SLC7A11; 1:1000, #A2413; ABclonal, Beijing, China), ferritin (dilution 1:2000, #T55648; Abmart, Shanghai, China), and β-actin (dilution 1:2000, #GB15003-100; Servicebio, Beijing, China).

### RNA isolation and RT-qPCR

Total RNA was extracted from heart tissues and H9C2 cells using Trizol reagent (#15596-026; Thermo Fisher Scientific, Waltham, USA). The extracted RNA was then reverse transcribed into cDNA using the Prime Script Reverse Transcriptase Kit (#RR047A; Takara, Dalian, China). The reverse transcription program was set as follows: 37°C for 15 min, 85°C for 5 s and 4°C for 4 min. TB Green Premix Ex Taq (#RR820A; Takara) was used for quantitative real-time PCR (RT-qPCR) performed on a T100 thermal cycler (Bio-Rad). Relative mRNA expression levels were analyzed using the 2 ^–△△Ct^ method, with *β-actin* serving as the endogenous control. All sequences of primers used are listed in Supplementary Table S1.

### Determination of MDA, GSH and LDH

The malondialdehyde (MDA), glutathione (GSH) and lactate dehydrogenase (LDH) levels in heart tissues and cellular supernatants from H9C2 cells were measured by spectrophotometry via an MDA assay kit (#BC0025; Solarbio, Beijing, China), a GSH assay kit (#BC1175; Solarbio) and an LDH assay kit (#A020-2-2; Nanjing Jiancheng Bioengineering Institute, Nanjing, China), respectively. All experiments were conducted in strict accordance with the manufacturer’s instructions.

### HE staining

The heart tissues were fixed in 4% paraformaldehyde and dehydrated in 50%, 75%, 85%, or 95% 100% ethanol. After dehydration, the tissues were embedded in paraffin and sliced into 5-μm-thick slices. After that, the slices were stained with hematoxylin and eosin for light microscopy.

### Masson’s trichrome staining

Heart tissue sections were deparaffinized for 1 h and hydrated in tap water or distilled water three times. The slices were subsequently stained with Weigert’s hematoxylin, acid ponceau and aniline blue via a Masson’s Trichrome Stain Kit (#G1340; Solarbio). All procedures strictly followed the manufacturer’s instructions, with the collagen fibers stained blue and the cytoplasmic smooth muscle fibers stained red.

### Transmission electron microscopy (TEM)

To observe alterations in mitochondrial morphology, TEM was conducted. Heart tissues were collected and cut into cubes measuring 1 mm × 1 mm × 1 mm. These tissue cubes were then immediately fixed in 2.5% glutaraldehyde at 4°C for 24 h. Following fixation, the tissue cubes underwent a series of steps, including dehydration, embedding, sectioning, staining and visualization. Finally, the stained sections were observed and imaged via a Hitachi HT7800 transmission electron microscope (Hitachi, Tokyo, Japan).

### Immunocytofluorescence staining

H9C2 cells were fixed with 4% paraformaldehyde for 20 min, permeabilized with 0.1% Triton X-100 for 10 min and blocked with 10% goat serum at room temperature for 30 min. Then, the cells were washed and stained with primary antibodies at 4°C for 15 h. The next day, the cells were washed three times with PBS and incubated with anti-rabbit IgG H&L (AF647, dilution 1:1000, #550048; Zenbio, Chengdu, China) secondary antibody at 37°C for 30 min, after which the nuclei were stained with DAPI for 6 min. A confocal laser scanning microscope (Leica, Wetzlar, Germany) was used for image acquisition. The primary antibodies used were as follows: HMOX1 (dilution 1:250, #ab189491; Abcam) and GPX4 (dilution 1:200, #ab125066; Abcam).

### ROS production assay

The level of ROS was determined with an ROS assay kit (#S0033S; Beyotime). DCFH-DA can be oxidized by ROS within cells to produce fluorescent DCF. Briefly, H9C2 cells were incubated with DCFH-DA at 37°C for 30 min, washed with PBS and replenished with fresh DMEM. Intracellular ROS were then immediately measured by detecting the fluorescence of the DCF byproduct with excitation at 488 nm and emission at 525 nm.

### Bioinformatics analysis

The raw data of the high-throughput transcriptome sequencing dataset GSE161052 were downloaded from the GEO database. This study included 6 C5BL/6J mice, with 3 mice in the control group and 3 mice in the DCM group. All ferroptosis-related genes were obtained from the FerrDb database. Raw counts were read in R, and DEGs were obtained using the DESeq2 algorithm. Next, ferroptosis-related DEGs were obtained, followed by GO analysis. Finally, the hub genes were identified using the Ctyohubba plugin.

### Lentiviral transfection

For *HMOX1* knockdown, H9C2 cells were transfected with a lentivirus synthesized by OBiO Technology Co., Ltd. (Shanghai, China), which carried the HMOX1 interference sequence or negative control. The interference sequence used was 5′-CCACACAGCACTACGTAAA-3′ and the negative control sequence was 5′-CCTAAGGTTAAGTCGCCCTCG-3′. The optimum multiplicity of infection (MOI) was 20 ( Supplementary Figure S1).

### Statistical analysis

All the statistical analyses were performed using GraphPad Prism 9.0.0 (GraphPad software, San Diego, USA). Data are presented as the mean ± SD. Unpaired Student’s *t* tests were performed for comparisons between two groups. One-way or two-way ANOVA with Sidak’s multiple comparison test was carried out for comparisons among multiple groups. *P*  < 0.05 was considered statistically significant.

## Results

### Establishment of a diabetic cardiomyopathy model

**Figure 1:**
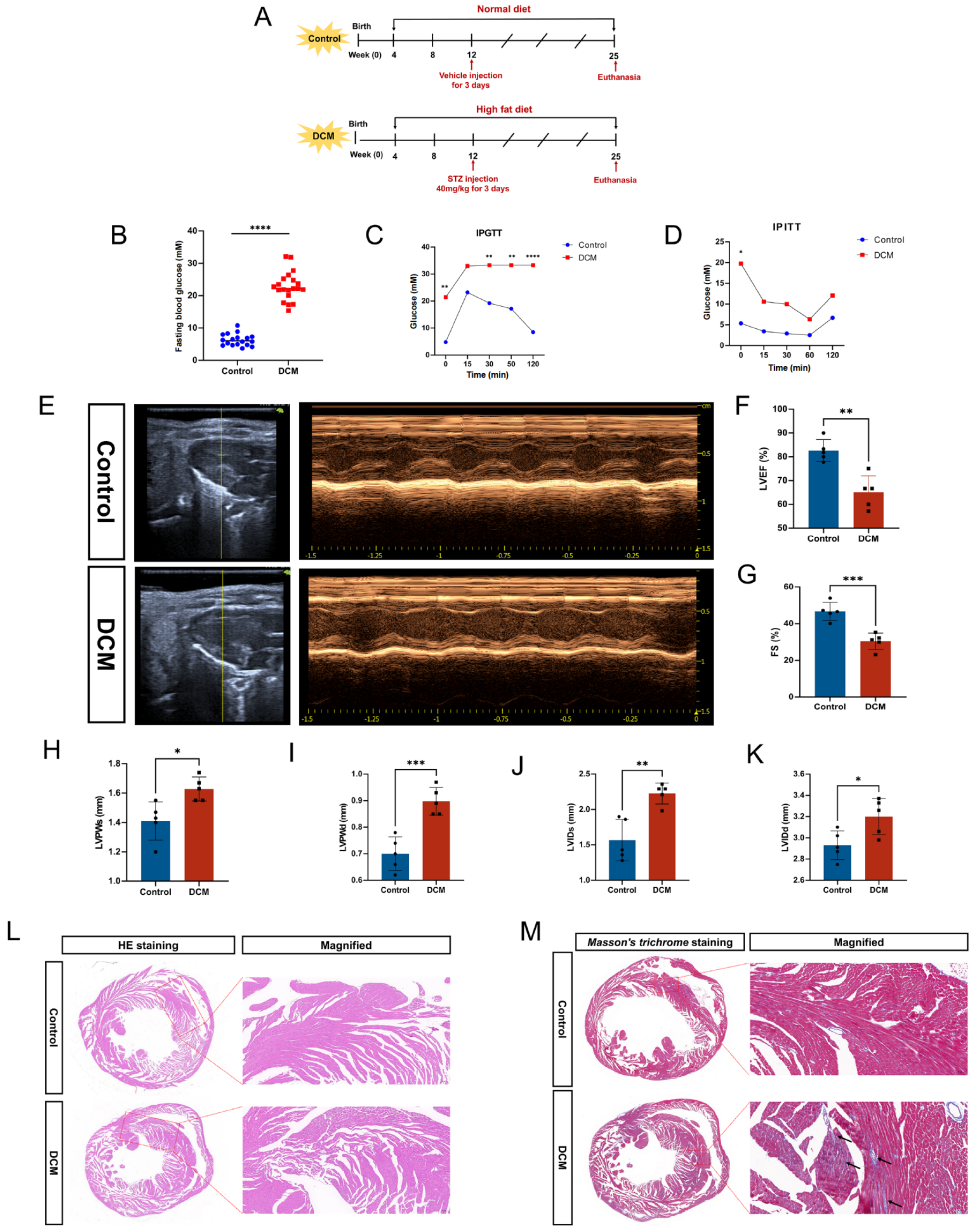
Figure 1 Construction of a diabetic cardiomyopathy mouse model (A) Experimental timeline of diabetic cardiomyopathy mouse model construction. (B) Fasting blood glucose levels of diabetic and control mice (n = 18–20 per group). (C) Intraperitoneal glucose (1 mg/g) tolerance test (IPGTT) for diabetic and control mice at 0, 15, 30, 60 and 120 min after injection (n = 5 per group). (D) Intraperitoneal insulin (1 U/kg) tolerance test (IPITT) for diabetic and control mice at 0, 15, 30, 60 and 120 min after injection (n = 5 per group). (E) Representative echocardiographic images of diabetic and control mice (two-dimensional echocardiogram was performed from the left ventricular long-axis view, and M-mode echocardiogram showed left ventricular dimensions). (F) Measurement of the left ventricular ejection fraction (LVEF) in diabetic and control mice. (G) Measurement of fractional shortening (FS) in diabetic and control mice (n = 5 per group). (H) Measurement of the systolic left ventricle posterior wall thickness (LVPW) in diabetic and control mice (n = 5 per group). (I) Measurement of diastolic left ventricle posterior wall thickness (LVPWd) in diabetic and control mice (n = 5 per group). (J) Measurement of the left ventricular internal dimension in systole (LVIDs) in diabetic and control mice (n = 5 per group). (K) Measurement of left ventricular internal dimension diastole (LVIDd) in diabetic and control mice (n = 5 per group). (L) Representative HE staining of diabetic and control hearts (scale bar: 100 μm). (M) Representative Masson’s trichrome staining of diabetic and control hearts (scale bar: 50 μm). *P < 0.05, **P < 0.01, ***P < 0.001, ****P < 0.0001. All the data were from 3–5 independent experiments.

We successfully established a diabetic cardiomyopathy model through high-fat diet feeding and intraperitoneal injection of low-dose streptozotocin ( Figure 1A). The fasting blood glucose levels were significantly greater in the diabetic group than in the control group ( Figure 1B). Furthermore, the blood glucose levels of diabetic mice were significantly elevated after glucose or insulin injection, in contrast to those of the control group ( Figure 1C,D). These findings indicate impaired glucose tolerance and insulin sensitivity in the diabetic group. Echocardiographic measurements ( Figure 1E) revealed that the LVEF and FS were lower in the diabetic group than in the control group ( Figure 1F,G). Additionally, parameters such as the LVPWs, LVPWd, LVIDs and LVIDd were significantly higher in diabetic mice ( Figure 1H–K). These results indicate that left ventricular remodeling and compromised systolic and diastolic function occur in diabetic mice. To investigate the pathogenesis of DCM, we performed HE staining and Masson’s trichrome staining on heart slices. HE staining revealed that cardiomyocytes in the control group were neatly arranged, with clear and dense structures and fewer extracellular interstitia. In contrast, cardiomyocytes in the diabetic group appeared disordered, with distorted and hypertrophied myocardial cells, increased cell-to-cell gaps, and increased perivascular extracellular interstitium ( Figure 1L). Moreover, Masson’s trichrome staining revealed prominent fibrosis in the hearts of diabetic mice compared with those of control mice ( Figure 1M). These results indicated that we successfully established an animal model of type 2 diabetic cardiomyopathy.

### The diabetic microenvironment induces heart ferroptosis *in vivo*

**Figure 2:**
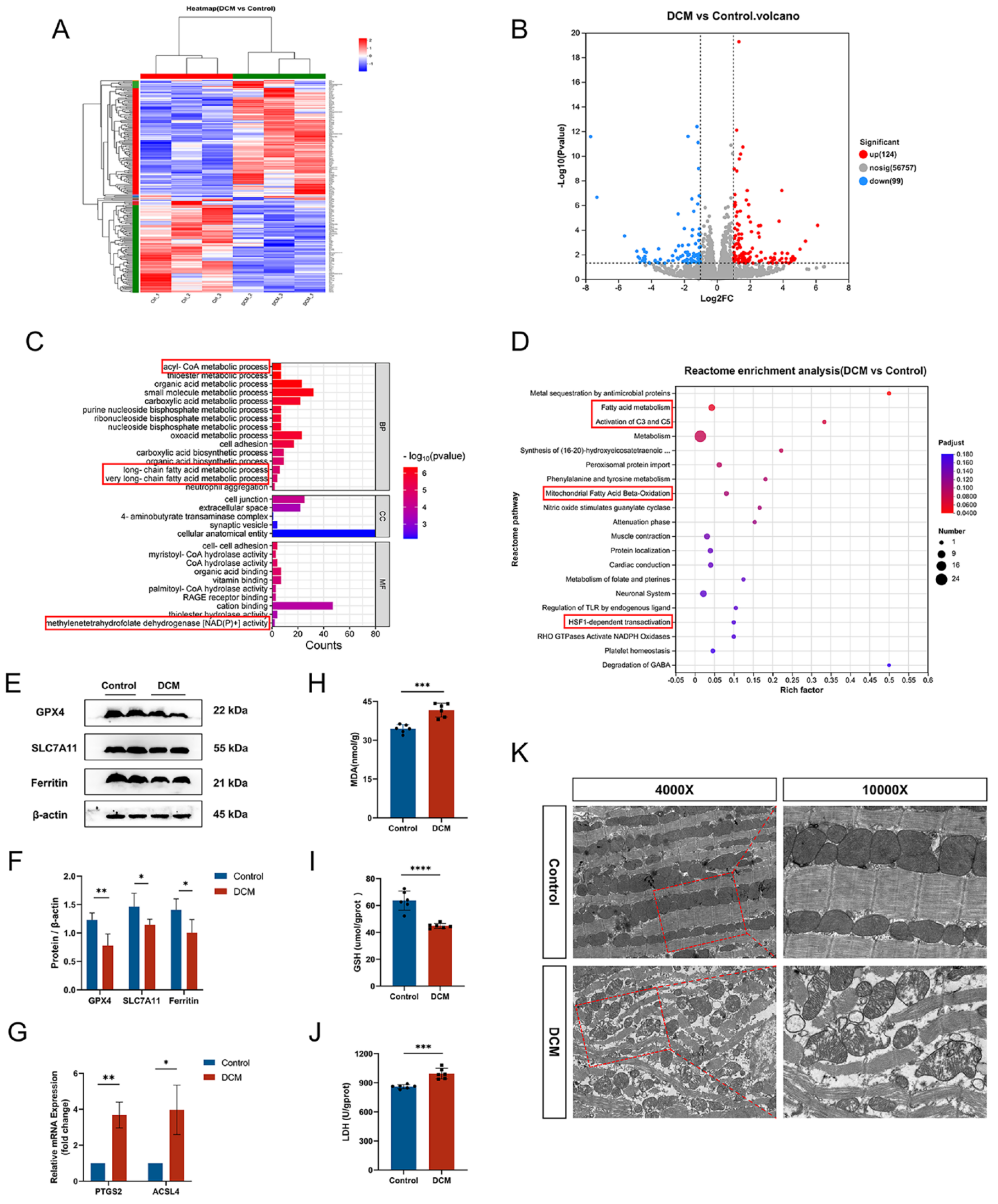
Figure 2 Diabetic microenvironment induces heart ferroptosis *in vivo* (A) Heatmap of differentially expressed genes (DEGs) between the diabetic and control groups (n = 3 per group). (B) Volcano plot of 223 DEGs between diabetic and control heart tissues; the inclusion criteria were a P value < 0.05 and log2FC > 1. The upregulated genes are represented by red dots, and the downregulated genes are represented by blue dots. (C) Gene ontology (GO, including biological process, cellular component, and molecular function) analysis of all DEGs. (D) Reactome enrichment analysis of all DEGs. (E) GPX4, SLC7A11, and ferritin protein expressions in diabetic and control heart tissues were detected by western blot analysis. (F) Quantitative analysis of GPX4, SLC7A11 and ferritin protein expression levels in the hearts of the two groups. (G) Quantitative analysis of ACSL4 and PTGS2 mRNA expressions in diabetic and control hearts. (H) Comparison of the MDA levels between the two groups. (I) GSH levels in the two groups. (J) LDH levels in the two groups. (K) Representative transmission electron microscopy (TEM) images of diabetic and control hearts. *P < 0.05, **P < 0.01, ***P < 0.001, ****P < 0.0001. All the data were from 3–6 independent experiments.

A DCM model was established, and heart tissues were collected for further investigation. To explore the underlying mechanisms of DCM, we compared the RNA sequences of heart tissues between the diabetic and control groups. The results revealed a total of 233 DEGs ( Figure 2A), with 124 genes upregulated and 99 genes downregulated ( Figure 2B). The inclusion criteria for DEGs were a *P* value < 0.05 and log _2_FC > 1. GO enrichment analysis revealed that the DEGs are enriched mainly in acyl-CoA metabolic processes, long-chain fatty acid metabolic processes and methylenetetrahydrofolate dehydrogenase [NAD(P) ^+^] activity ( Figure 2C). Reactome enrichment analysis revealed that the DEGs are involved primarily in fatty acid metabolism, activation of C3 and C5, and mitochondrial fatty acid beta-oxidation ( Figure 2D). These processes are closely linked to iron homeostasis and ferroptosis, suggesting that the diabetic microenvironment may activate ferroptosis-related metabolic pathways in heart tissues.

Furthermore, western blot analysis revealed lower protein expressions of GPX4, SLC7A11 and ferritin in the diabetic group ( Figure 2E,F), whereas RT-qPCR revealed significantly higher mRNA expressions of prostaglandin-endoperoxide synthase 2 ( *PTGS2*) and acyl-CoA synthetase long chain family member 4 ( *ACSL4*) (two acknowledged markers of ferroptosis [13]) in the diabetic group than in the control group ( Figure 2G). Elevated levels of malondialdehyde (MDA), a final product of lipid peroxidation closely associated with ferroptotic cell death, were observed in the heart tissues of the diabetic group ( Figure 2H). Additionally, GSH, an important component for cellular antioxidant defense and a necessary cofactor of GPX4, was decreased in diabetic heart tissues ( Figure 2I). Conversely, LDH level, which is indicative of cytotoxicity, was elevated in the diabetic group ( Figure 2J). TEM images revealed distinct differences between the control and DCM groups. In the control group, the myocardial fibers presented abundant myofilaments and well-arranged z-lines. The mitochondria appeared linear with regular shapes, intact structures, and abundant, intact mitochondrial cristae without vacuolation. However, in the DCM group, the nuclei appeared wrinkled, the mitochondria exhibited shrinkage and degeneration with increased membrane density, the mitochondrial cristae fractured or disappeared, the myonuclei lost their normal structure, and the intercalated disc gaps widened, fractured and lysed ( Figure 2K). These findings collectively suggest the involvement of ferroptosis in the pathogenesis of DCM.

### GP treatment activates ferroptosis in H9C2 cells

**Figure 3:**
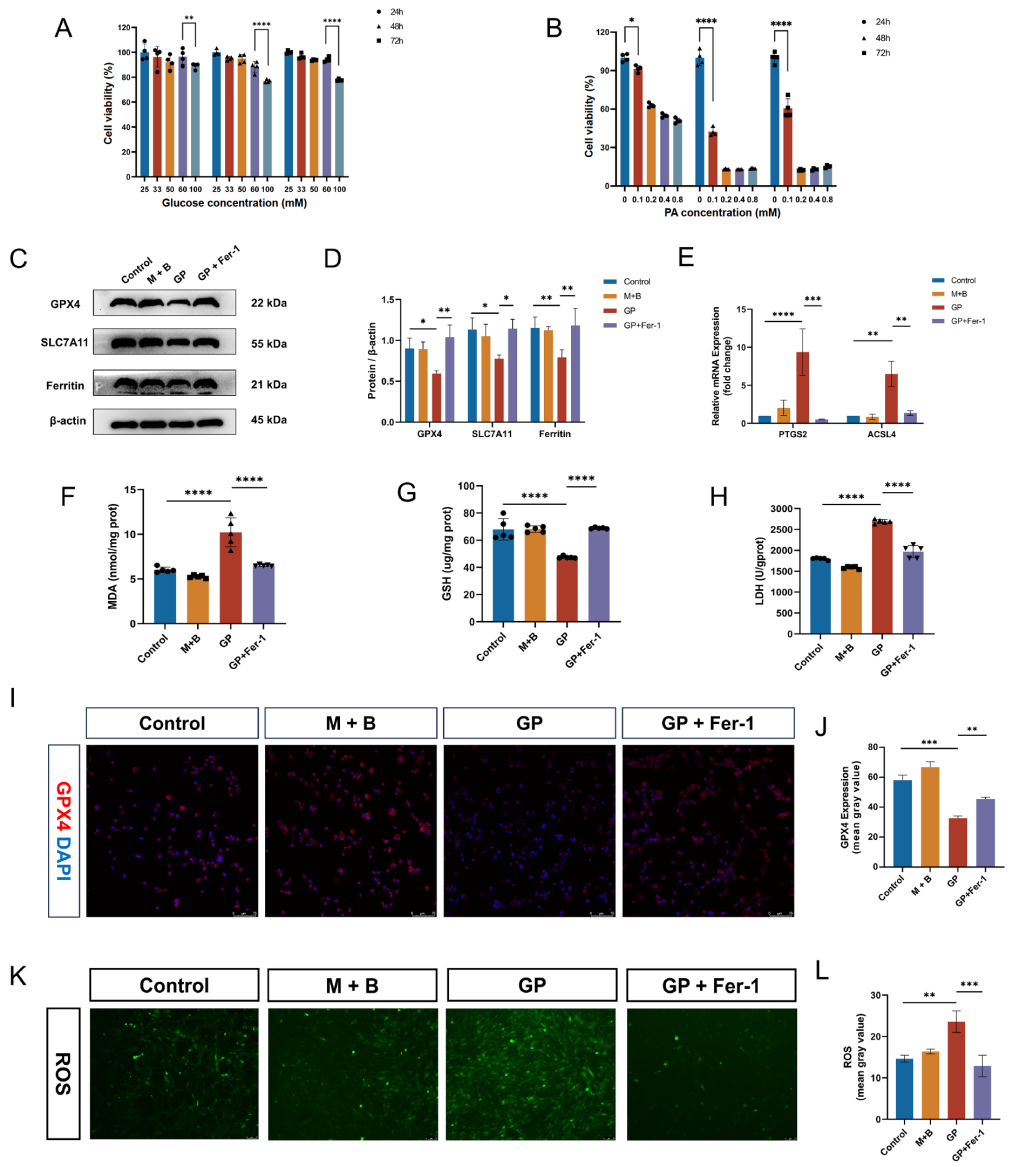
Figure 3 High glucose and palmitic acid treatment activates ferroptosis *in vitro* (A) Cell viability of H9C2 cells treated with 25, 33, 50, 60, or 100 mM glucose for 24 h, 48 h, or 72 h, respectively. (B) Viability of H9C2 cells treated with 25, 33, 50, 60, or 100 mM palmitic acid for 24, 48, or 72 h, respectively. (C) GPX4, SLC7A11, and ferritin protein expressions in H9C2 cells in the control, M + B, GP, and GP + Fer-1 groups. (D) Quantitative analysis of GPX4, SLC7A11 and ferritin protein expression levels in H9C2 cells in the four groups. (E) Quantitative analysis of ACSL4 and PTGS2 mRNA expressions in H9C2 cells in the four groups. (F) MDA levels of H9C2 cells in the control, M + B, GP, and GP + Fer-1 groups. (G) GSH levels in H9C2 cells in the four groups. (H) LDH levels in H9C2 cells in the four groups. (I) Representative images of immunofluorescence staining of GPX4 in the four groups (scale bar: 75 μm). (J) Quantification of immunofluorescence staining for GPX4 in the four groups. (K) Representative images of intracellular ROS in the four groups (scale bar: 75 μm). (L) Quantification of the intracellular ROS levels in the four groups. *P < 0.05, **P < 0.01, ***P < 0.001, ****P < 0.0001. All the data were from 3–6 independent experiments.

To further confirm the induction of ferroptosis by the diabetic microenvironment *in vitro*, we subjected H9C2 cells to normal glucose, mannitol/BSA and high glucose/palmitic acid. First, we assessed the impact of various concentrations of glucose and palmitic acid on H9C2 cell viability. CCK-8 assay revealed that cell viability was decreased at 100 mM glucose compared with 33 mM, 50 mM, and 60 mM glucose at 24, 48 and 72 h ( Figure 3A). Similarly, palmitic acid at concentrations of 0.1, 0.2, 0.4, and 0.8 mM gradually decreased cell viability in a time-dependent manner ( Figure 3B). On the basis of these results, we determined that 60 mM glucose and 0.1 mM palmitic acid were the optimal concentrations for subsequent experiments. In addition, we observed a gradual decrease in GPX4 protein expression at 6, 12, 24 and 48 h ( Supplementary Figure S2), indicating the progressive aggravation of ferroptosis with prolonged drug exposure. Therefore, we selected a 48-h incubation time for subsequent cell experiments.

Western blot analysis revealed reduced protein expressions of GPX4, SLC7A11 and ferritin in the GP group, but these effects were effectively reversed by Fer-1 ( Figure 3C,D). The RT-qPCR results revealed elevated mRNA expressions of *PTGS2* and *ACSL4* with GP treatment, which was reversed after the administration of Fer-1 ( Figure 3E). The levels of MDA and LDH were significantly increased in the GP group, whereas the GSH level was decreased. However, treatment with 10 μM Fer-1 (a definitive ferroptosis inhibitor [14]) led to decreased MDA and LDH levels and increased GSH level ( Figure 3F–H). Immunofluorescence staining revealed decreased GPX4 expression and increased intracellular ROS levels in the GP group ( Figure 3I–L). However, GPX4 expression was elevated and ROS levels were decreased after Fer-1 treatment. These results indicated that the diabetic microenvironment could induce ferroptosis in H9C2 cells, and this effect could be reversed by Fer-1.

### HMOX1 expression is elevated in the diabetic microenvironment

**Figure 4:**
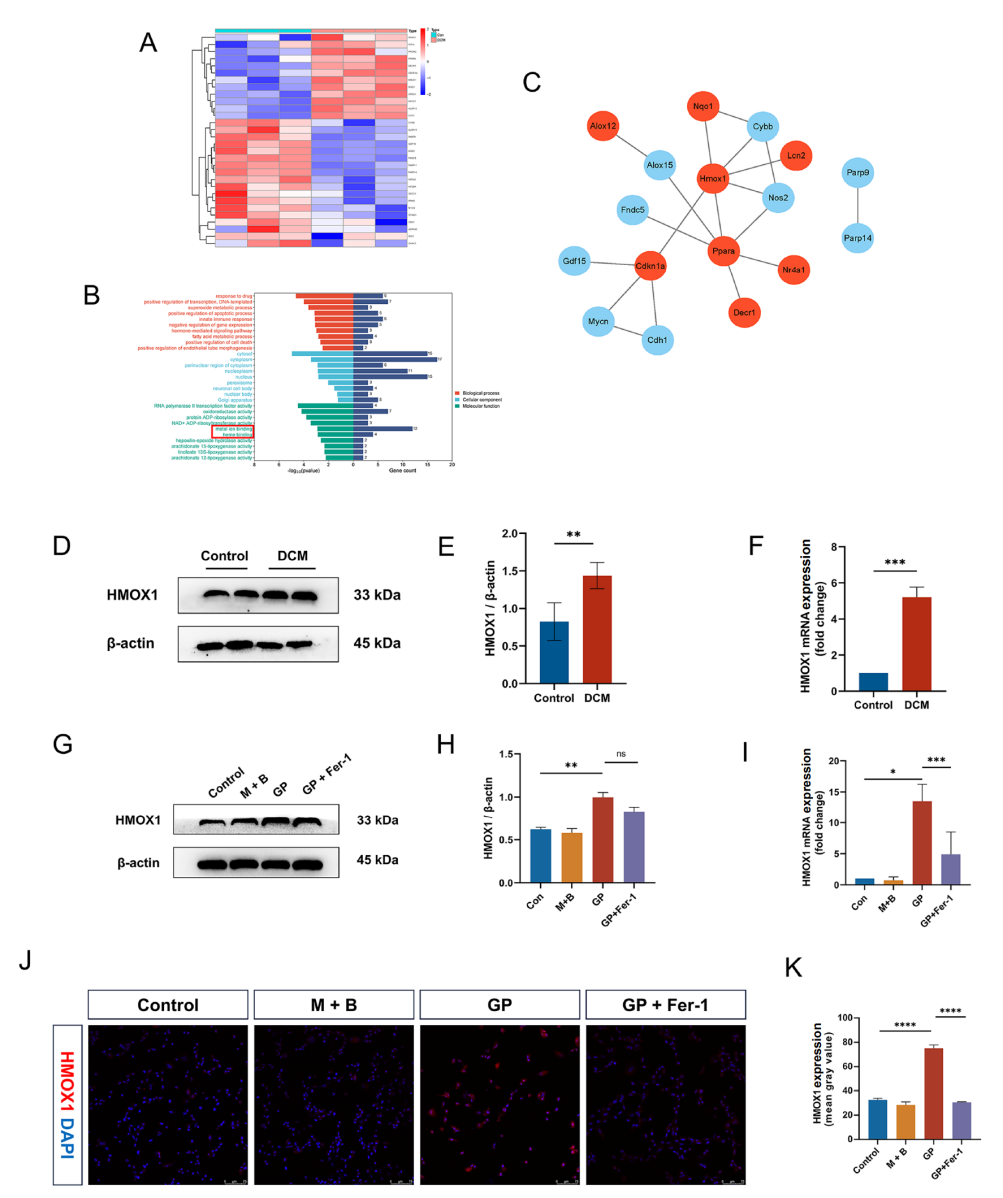
Figure 4 Increased HMOX1 expression in diabetic hearts and GP-treated H9C2 cells (A) Heatmap of DEGs in the GSE161052 dataset between the diabetic and control groups. (B) GO enrichment analysis of all DEGs in GSE161052. (C) HMOX1 was identified as a hub gene via the Cytohubba plugin. (D) HMOX1 protein expressions in heart tissues from diabetic and control mice were determined via western blot analysis. (E) Quantitative analysis of HMOX1 protein expression levels in diabetic and control hearts. (F) Quantitative analysis of HMXO1 mRNA expression in diabetic and control hearts. (G) HMOX1 protein expressions in H9C2 cells in the control, M + B, GP, and GP + Fer-1 groups. (H) Quantitative analysis of HMOX1 protein expression levels in the four groups. (I) Quantitative analysis of HMOX1 mRNA expressions in H9C2 cells in the control, M + B, GP, and GP + Fer-1 groups. (J) Representative images of immunofluorescence staining of HMOX1 in the four groups (scale bar: 75 μm). (K) Quantification of immunofluorescence staining of HMOX1 in the four groups. All the data were from 3–6 independent experiments.

HMOX1, a cell-induced oxidative stress modulator, decomposes heme into carbon monoxide, biliverdin and ferrous iron [15]. Therefore, we hypothesized that HMOX1 may be responsible for diabetes-induced ferroptosis. Thus, we investigated HMOX1 expression in the diabetic microenvironment. First, bioinformatics analysis revealed that DEGs between control and diabetic heart tissues were significantly enriched in metal ion binding and heme binding ( Figure 4A,B), and HMOX1 was identified as the most significant hub gene ( Figure 4C). The western blot analysis and RT-qPCR results revealed significantly higher levels of HMOX1 in diabetic mice than in control mice ( Figure 4D–F). Moreover, in H9C2 cells, GP treatment increased HMOX1 expression, which was reversed by Fer-1 ( Figure 4G–I). Immunofluorescence staining also revealed elevated HMOX1 expression in the GP-treated group compared with the control group ( Figure 4J,K). In conclusion, HMOX1 expression was elevated in the diabetic microenvironment both *in vivo* and *in vitro*.

### HMOX1 is essential for diabetes-induced ferroptosis

**Figure 5:**
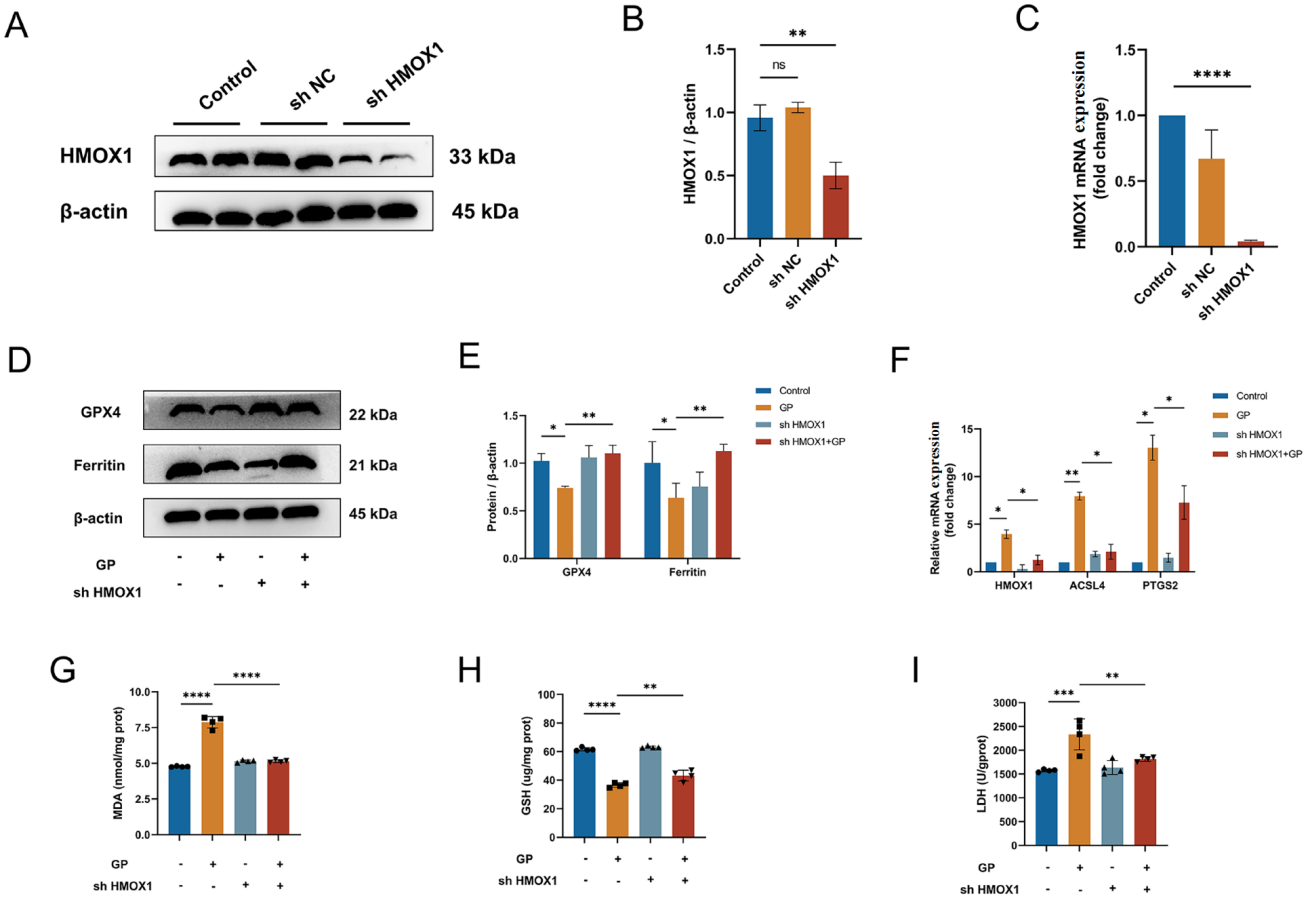
Figure 5 Knockdown of *HMXO1* inhibits ferroptosis in H9C2 cells (A) HMOX1 protein expression was detected by western blot analysis in H9C2 cells transfected with lentivirus carrying the HMOX1 interference sequence or negative control. (B) Quantitative analysis of HMOX1 protein expression level in H9C2 cells after lentivirus transfection. (C) Quantitative analysis of HMOX1 mRNA expression in H9C2 cells after lentivirus transfection. (D) GPX4 and ferritin protein expressions were determined by western blot analysis. (E) Quantitative analysis of GPX4 and ferritin protein expression levels in H9C2 cells in the control, GP, shHMOX1, and GP + shHMOX1 groups. (F) Quantitative analysis of HMOX1, ACSL4, and PTGS2 mRNA expressions in the control, GP, shHMOX1, and GP + shHMOX1 groups. (G) MDA levels in H9C2 cells in the control, GP, shHMOX1, and GP + shHMOX1 groups. (H) GSH levels in H9C2 cells in the four groups. (I) LDH levels in H9C2 cells in the four groups. nP > 0.05, *P < 0.05, **P < 0.01, ***P < 0.001, ****P < 0.0001. All the data were from 3–6 independent experiments.

Next, H9C2 cells were transfected with a lentivirus carrying an interference sequence for HMOX1 or a negative control. As expected, both HMOX1 protein and mRNA expression levels were downregulated following lentivirus transfection ( Figure 5A–C). Western blot analysis revealed a decrease in GPX4 and ferritin protein expression with GP treatment, whereas the transfection of HMOX1-depleted lentivirus increased the expressions of GPX4 and ferritin ( Figure 5D,E). RT-qPCR results demonstrated that knockdown of *HMOX1* rescued the upregulation of PTGS2 and ACSL4 in H9C2 cells treated with GPs ( Figure 5F). Similar trends were also observed in the levels of MDA, GSH and LDH ( Figure 5G–I). Therefore, inhibition of HMOX1 attenuated glycolipid toxicity and reduced ferroptosis in H9C2 cells.

### Targeting HMOX1 or ferroptosis alleviates diabetic cardiomyopathy

**Figure 6:**
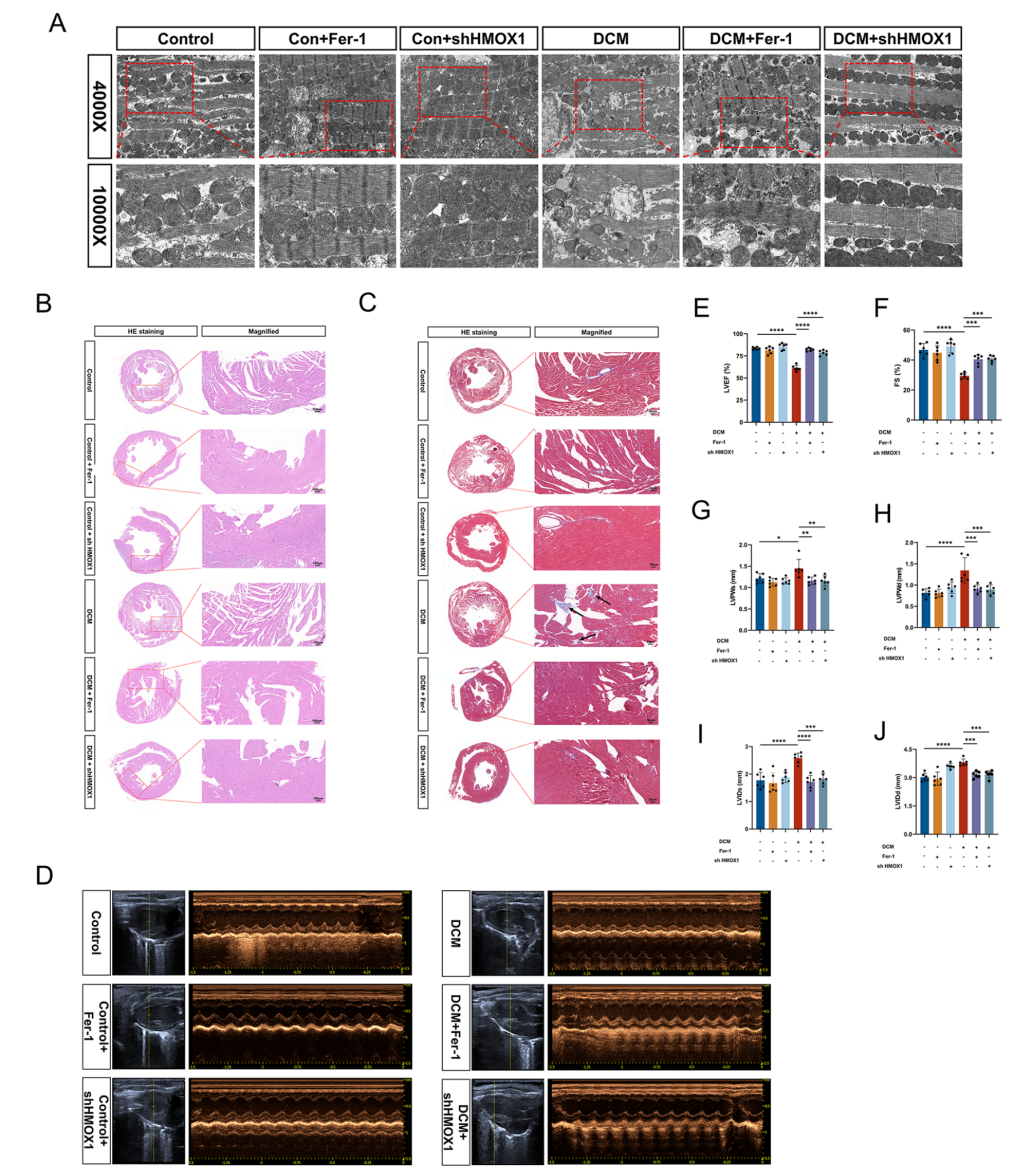
Figure 6 Knockdown of *HMOX1* alleviates DCM *in vivo* (A) Representative TEM images of the control and DCM groups treated with Fer-1 or HMOX1 AAV. (B) Representative images of HE staining in all groups. (C) Representative images of Masson’s trichrome staining in all groups. (D) Representative echocardiographic images of all groups. (E–J) Measurements of the LVEF, FS, LVPWs, LVPWd, LVIDs, and LVIDd in all groups (n = 6 each group). *P < 0.05, **P < 0.01, ***P < 0.001, ****P < 0.0001. All the data were from 3–6 independent experiments.

To validate the role of HMOX1 in DCM *in vivo*, we administered HMOX1 AAV9 and Fer-1 to diabetic mice. The TEM results revealed that mitochondrial morphology was significantly rescued by HMOX1 AAV9 and Fer-1 ( Figure 6A). In addition, histological examinations via HE and Masson’s trichrome staining revealed the restoration of myocardial morphology and reduction in fibrosis ( Figure 6B,C). Furthermore, echocardiographic images demonstrated that cardiac function was significantly improved following the inhibition of HMOX1 and ferroptosis ( Figure 6D–J). These results indicated that intervention with HMOX1 expression reduced ferroptosis, improved cardiac function, and alleviated diabetic cardiomyopathy.

## Discussion

Diabetes has emerged as a global epidemic that poses a significant threat to public health. The development of DM and its complications involve a range of pathological processes, including lipid oxidative stress and inflammatory activation [11]. DCM is increasingly recognized as a global disease, contributing to heart failure episodes in at least 50% of diabetic patients [16]. However, current medications and therapeutic approaches that focus primarily on optimal glycemic control have proven inadequate in addressing this issue. Therefore, identifying potential molecular mechanisms and novel therapeutic targets is imperative. Research indicates that ferroptosis plays a pivotal role in the pathogenesis of diabetes and its associated complications [17]. In this study, we successfully established a DCM mouse model and observed distinct ferroptotic phenotypes in heart tissues characterized by deactivation of the antioxidant system, upregulation of ferroptosis-related genes, lipid peroxidation, ROS accumulation, and morphological alterations in mitochondria. Furthermore, we established an *in vitro* diabetic model using H9C2 cells treated with high glucose and palmitic acid to evaluate the occurrence of ferroptosis. Notably, our findings highlight the essential role of HMOX1 in diabetic ferroptosis. Specifically, *HMOX1* knockdown reversed ferroptosis in the diabetic microenvironment, suggesting potential therapeutic benefits in alleviating DCM.

**Figure 7:**
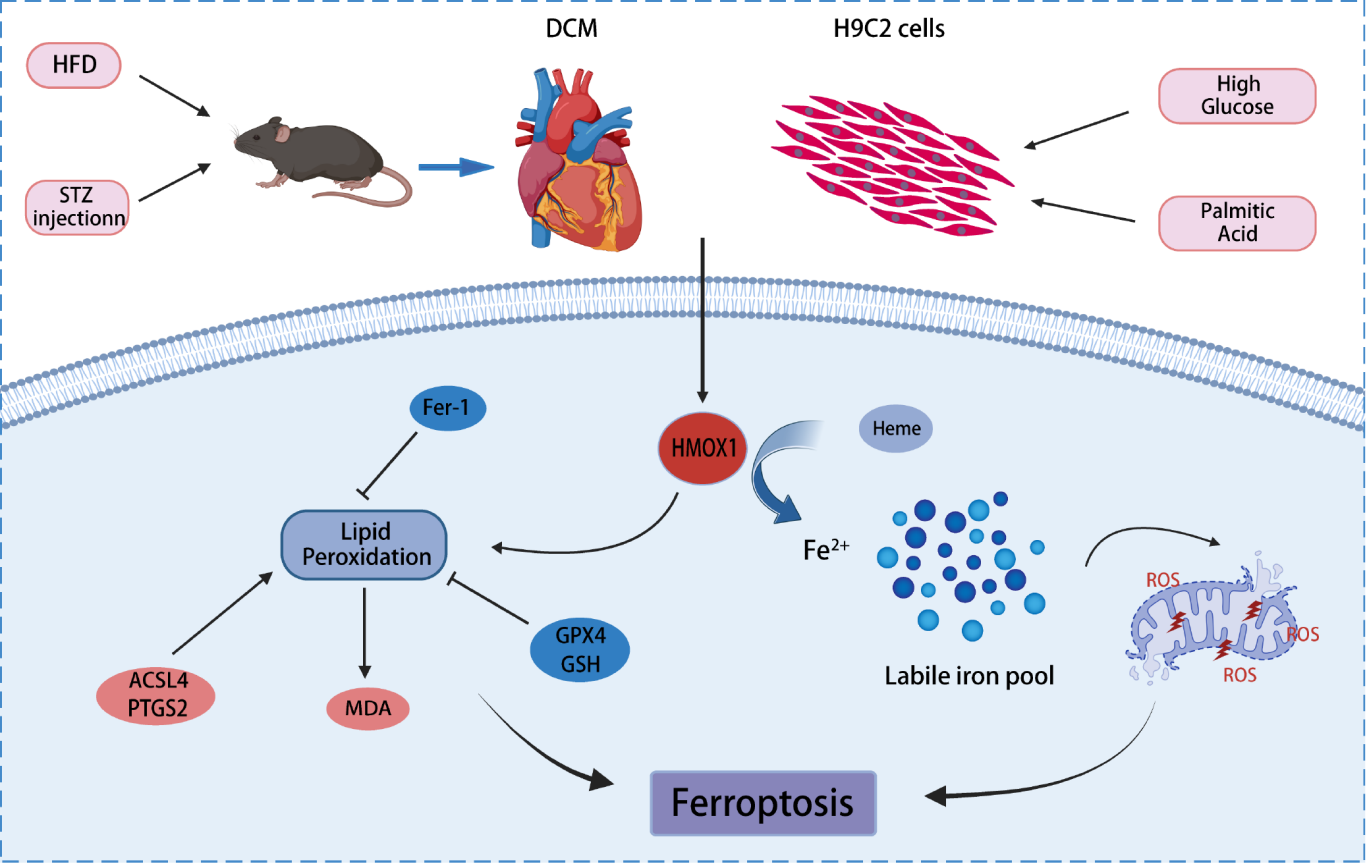
Figure 7 Schematic illustration highlighting the central role of HMOX1 in inducing ferroptosis in diabetic cardiomyopathy Ferroptosis was induced and HMOX1 expression was upregulated both in vivo and in vitro under diabetic microenvironment. The increased HMOX1 can break down heme into biliverdin, carbon monoxide and iron ions, which can exacerbate lipid peroxidation and lead to ferroptosis. Knockdown of HMOX1 alleviates diabetic cardiomyopathy through inhibiting lipid peroxidation and ferroptosis.

Ferroptosis is a unique form of cell death that differs from traditional apoptosis and necroptosis in terms of morphology and mechanism [18]. Iron plays crucial roles as both an essential cofactor in enzymatic metabolic processes and a catalyst of poorly controlled redox-cycling reactions, making it a double-edged sword [19]. Excessive iron accumulation can lead to intracellular ROS buildup and trigger the Fenton reaction [20]. Ferroptosis activation is associated with abnormal iron metabolism and lipid peroxidation, along with inactivation of Systems Xc ^-^ and GPX4. Fer-1, a synthetic antioxidant, acts as a potent scavenger of hydrogen peroxy radicals and exerts antiferroptotic effects similar to those of GPX4 [21]. Research has shown that Fer-1 has no effect on glucose metabolism but can alleviate HFD-induced lipid metabolism disorders in diabetic mice with atherosclerosis [22]. In our study, Fer-1 reversed microenvironment-induced ferroptosis in H9C2 cells, reduced cardiac fibrosis and improved cardiac function. In conclusion, ferroptosis may represent a new therapeutic target for DCM ( Figure 7).

DCM increases the risk of heart failure independent of hypertension and coronary heart disease [23]. Studies have demonstrated the involvement of ferroptosis in the progression of diabetes complications. The high-glucose microenvironment promotes changes in upstream regulatory factors, leading to iron overload and diminished antioxidant capacity [24]. Among these factors, the Nrf2 signaling pathway plays a particularly significant role in diabetes-induced ferroptosis [ 25, 26]. Evidence has revealed the presence of typical ferroptotic characteristics in DCM hearts. A study published in 2022 demonstrated that ferroptosis is evident in hearts affected by DCM and that this process can be prevented by sulforaphane via the AMPK/NRF2 pathway [27]. Another study confirmed that canagliflozin mitigated ferroptosis and improved myocardial oxidative stress in mice with DCM [28]. Additionally, 6-gingerol was found to alleviate cardiomyocyte ferroptosis and inflammation in both DCM- and high glucose-induced H9C2 cells [29]. These findings are consistent with our experimental results.

Among human diabetes patients, 90% have type 2 diabetes, and most individuals exhibit insulin resistance in addition to impaired insulin secretion. In 2000, Reaven and colleagues established a type 2 diabetes model by feeding nonobese inbred rats with a high-fat diet and injecting STZ [30]. In our study, we established a type 2 diabetes mouse model. Echocardiography confirmed the presence of DCM in 25-week-old mice. We further validated the occurrence of ferroptosis in DCM hearts, accompanied by changes in mitochondrial morphology. Previous studies have indicated that in type 2 diabetic mice, the levels of free fatty acids (FFAs), particularly palmitic acid (PA), are significantly elevated. These findings suggest that high glucose and FFA levels are key characteristics of the diabetic microenvironment. It has been reported that PA is the most abundant saturated FFA [31]. Therefore, in subsequent cell experiments, we simulated the diabetic microenvironment by exogenously adding glucose and PA to H9C2 cells.

HMOX1 is a crucial enzyme involved in heme catabolism, catalyzing the degradation of heme into iron, carbon monoxide and biliverdin [32]. HMOX1 is known to play crucial roles in various diseases, such as cardiovascular conditions [33], diabetes, cancer [34], and neurodegenerative disorders [35]. In addition, HMOX1 protects cells by regulating oxidative stress [36], apoptosis [37], and inflammatory responses [38]. In recent years, the role of HMOX1 in ferroptosis has been extensively studied, yielding controversial findings. Researchers have identified HMOX1 as a key gene associated with ferroptosis in atherosclerosis [39] ^,^ and its upregulation promoted ferroptosis in diabetic atherosclerosis [22]. Another study revealed notable upregulation of HMOX1 in ferroptotic osteocytes associated with diabetic osteoporosis [40]. However, one study reported that melatonin significantly reduces ferroptosis level by activating the Nrf2/HO-1 pathway [41]. Moreover, inhibition of the Keap-Nrf2/HO-1 pathway significantly abolished the antiferroptotic and anti-inflammatory effects of PX in LPS-treated cells [42].

In our study, we observed the activation of ferroptosis in DCM heart tissues. Interestingly, the RNA sequencing results did not reveal any difference in HMOX1 expression between diabetic and control heart tissues. However, the western blot analysis and RT-qPCR results demonstrated significant upregulation of HMOX1 expression in the diabetic microenvironment both *in vivo* and *in vitro*. Bioinformatics analysis also revealed that HMOX1 expression is upregulated and that HMOX1 is a hub gene of ferroptosis. This discrepancy may be attributed to insufficient biological replicates of sequenced samples and individual variability. Furthermore, our findings indicate that inhibition of HMOX1 helps rescue ferroptosis induced by high glucose and palmitic acid. In summary, HMOX1 plays an essential role in ferroptosis and has diverse functions in different diseases. Previous studies reported that HMOX1 expression is induced by activation of the redox-sensitive transcription factors Nrf2 and HIF-1α [ 14, 43]. The NRF2:c-JUN heterodimer is the upstream activator of *HMXO1* transcription in the diabetic microenvironment [40]. Therefore, further research is needed to understand the direct interactions between HMOX1 and other factors influencing ferroptosis in DCM.

Nevertheless, our study has several limitations that should be acknowledged. First, we only conducted *HMOX1*-knockdown experiments and did not investigate the effect of HMOX1 overexpression on ferroptosis. Second, the interactions between HMOX1 and classical ferroptosis biomarkers (GPX4, SLC7A11, and ACSL4) remain unclear. In future studies, we will seek further validation in this area.

In summary, our study provides valuable insights into the role of ferroptosis in the pathogenesis of diabetic cardiomyopathy. We demonstrated the activation of HMOX1 in the diabetic microenvironment both *in vivo* and *in vitro*. These findings suggest that targeting ferroptosis and HMOX1 may serve as novel mechanisms and potential therapeutic targets for the prevention and treatment of DCM. Further research focusing on the clinical applications of this strategy is warranted.

## Supporting information

24696Supplementary_data

## Supplementary Data

Supplementary data is available at *Acta Biochimica et Biophysica Sinica* online.

## References

[1] The L (2017). Diabetes: a dynamic disease. Lancet.

[2] Glovaci D, Fan W, Wong ND (2019). Epidemiology of diabetes mellitus and cardiovascular disease. Curr Cardiol Rep.

[3] Ke J, Pan J, Lin H, Gu J (2023). Diabetic cardiomyopathy: a brief summary on lipid toxicity. ESC Heart Fail.

[4] Dillmann WH (2019). Diabetic cardiomyopathy. Circ Res.

[5] Jia G, Whaley-Connell A, Sowers JR (2018). Diabetic cardiomyopathy: a hyperglycaemia- and insulin-resistance-induced heart disease. Diabetologia.

[6] Stockwell BR (2022). Ferroptosis turns 10: emerging mechanisms, physiological functions, and therapeutic applications. Cell.

[7] Jiang X, Stockwell BR, Conrad M (2021). Ferroptosis: mechanisms, biology and role in disease. Nat Rev Mol Cell Biol.

[8] Cao JY, Dixon SJ (2016). Mechanisms of ferroptosis. Cell Mol Life Sci.

[9] Stockwell BR, Friedmann Angeli JP, Bayir H, Bush AI, Conrad M, Dixon SJ, Fulda S (2017). Ferroptosis: a regulated cell death nexus linking metabolism, redox biology, and disease. Cell.

[10] Wang K, Chen XZ, Wang YH, Cheng XL, Zhao Y, Zhou LY, Wang K (2022). Emerging roles of ferroptosis in cardiovascular diseases. Cell Death Discov.

[11] Altamura S, Kopf S, Schmidt J, Müdder K, da Silva AR, Nawroth P, Muckenthaler MU (2017). Uncoupled iron homeostasis in type 2 diabetes mellitus. J Mol Med.

[12] Shou Y, Li X, Fang Q, Xie A, Zhang Y, Fu X, Wang M (2024). Progress in the treatment of diabetic cardiomyopathy, a systematic review. Pharmacol Res Perspec.

[13] Tang D, Chen X, Kang R, Kroemer G (2021). Ferroptosis: molecular mechanisms and health implications. Cell Res.

[14] Miotto G, Rossetto M, Di Paolo ML, Orian L, Venerando R, Roveri A, Vučković AM (2020). Insight into the mechanism of ferroptosis inhibition by ferrostatin-1. Redox Biol.

[15] Menon AV, Liu J, Tsai HP, Zeng L, Yang S, Asnani A, Kim J (2022). Excess heme upregulates heme oxygenase 1 and promotes cardiac ferroptosis in mice with sickle cell disease. Blood.

[16] Evangelista I, Nuti R, Picchioni T, Dotta F, Palazzuoli A (2019). Molecular dysfunction and phenotypic derangement in diabetic cardiomyopathy. Int J Mol Sci.

[17] Yang XD, Yang YY (2022). Ferroptosis as a novel therapeutic target for diabetes and its complications. Front Endocrinol.

[18] Dixon SJ, Lemberg KM, Lamprecht MR, Skouta R, Zaitsev EM, Gleason CE, Patel DN (2012). Ferroptosis: an iron-dependent form of nonapoptotic cell death. Cell.

[19] Stoyanovsky DA, Tyurina YY, Shrivastava I, Bahar I, Tyurin VA, Protchenko O, Jadhav S (2019). Iron catalysis of lipid peroxidation in ferroptosis: regulated enzymatic or random free radical reaction?. Free Radical Biol Med.

[20] He YJ, Liu XY, Xing L, Wan X, Chang X, Jiang HL (2020). Fenton reaction-independent ferroptosis therapy via glutathione and iron redox couple sequentially triggered lipid peroxide generator. Biomaterials.

[21] Hofmans S, Berghe TV, Devisscher L, Hassannia B, Lyssens S, Joossens J, Van Der Veken P (2016). Novel ferroptosis inhibitors with improved potency and ADME properties. J Med Chem.

[22] Meng Z, Liang H, Zhao J, Gao J, Liu C, Ma X, Liu J (2021). HMOX1 upregulation promotes ferroptosis in diabetic atherosclerosis. Life Sci.

[23] Murtaza G, Virk HUH, Khalid M, Lavie CJ, Ventura H, Mukherjee D, Ramu V (2019). Diabetic cardiomyopathy——A comprehensive updated review. Prog Cardiovasc Dis.

[24] Cundy T, Holden A, Stallworthy E (2021). Early worsening of diabetic nephropathy in type 2 diabetes after rapid improvement in chronic severe hyperglycemia. Diabetes Care.

[25] Wu Y, Zhao Y, Yang H, Wang Y, Chen Y (2021). HMGB1 regulates ferroptosis through Nrf2 pathway in mesangial cells in response to high glucose. Biosci Rep.

[26] Sun X, Wang X, Zhao Z, Chen J, Li C, Zhao G (2020). Paeoniflorin accelerates foot wound healing in diabetic rats though activating the Nrf2 pathway. Acta Histochemica.

[27] Wang X, Chen X, Zhou W, Men H, Bao T, Sun Y, Wang Q (2022). Ferroptosis is essential for diabetic cardiomyopathy and is prevented by sulforaphane via AMPK/NRF2 pathways. Acta Pharm Sin B.

[28] Du S, Shi H, Xiong L, Wang P, Shi Y (2022). Canagliflozin mitigates ferroptosis and improves myocardial oxidative stress in mice with diabetic cardiomyopathy. Front Endocrinol.

[29] Wu S, Zhu J, Wu G, Hu Z, Ying P, Bao Z, Ding Z (2022). 6-Gingerol alleviates ferroptosis and inflammation of diabetic cardiomyopathy via the Nrf2/HO-1 pathway. Oxid Med Cell Longev.

[30] Reed MJ, Meszaros K, Entes LJ, Claypool MD, Pinkett JG, Gadbois TM, Reaven GM (2000). A new rat model of type 2 diabetes: the fat-fed, streptozotocin-treated rat. Metabolism.

[31] Al Saedi A, Myers DE, Stupka N, Duque G (2020). 1,25(OH)2D3 ameliorates palmitate-induced lipotoxicity in human primary osteoblasts leading to improved viability and function. Bone.

[32] Liu R, Zhang X, Nie L, Sun S, Liu J, Chen H (2023). Heme oxygenase 1 in erythropoiesis: an important regulator beyond catalyzing heme catabolism. Ann Hematol.

[33] Ryter SW (2022). Heme oxygenase-1: an anti-inflammatory effector in cardiovascular, lung, and related metabolic disorders. Antioxidants.

[34] Hassannia B, Vandenabeele P, Vanden Berghe T (2019). Targeting ferroptosis to iron out cancer. Cancer Cell.

[35] Facchinetti MM (2020). Heme-oxygenase-1. Antioxid Redox Signal.

[36] Morgenstern C, Lastres-Becker I, Demirdöğen BC, Costa VM, Daiber A, Foresti R, Motterlini R (2024). Biomarkers of NRF2 signalling: current status and future challenges. Redox Biol.

[37] Al-Owais MM, Dallas ML, Boyle JP, Scragg JL, Peers C. Heme oxygenase-1 influences apoptosis via CO-mediated inhibition of K
^+^ channels.
Adv Exp Med Biol 2015, 860: 343–351. https://doi.org/10.1007/978-3-319-18440-1_39.

[38] Rushworth SA, O′Connell MA (2004). Haem oxygenase-1 in inflammation. Biochem Soc Trans.

[39] Wu D, Hu Q, Wang Y, Jin M, Tao Z, Wan J (2022). Identification of HMOX1 as a critical ferroptosis-related gene in atherosclerosis. Front Cardiovasc Med.

[40] Yang Y, Lin Y, Wang M, Yuan K, Wang Q, Mu P, Du J (2022). Targeting ferroptosis suppresses osteocyte glucolipotoxicity and alleviates diabetic osteoporosis. Bone Res.

[41] Ma H, Wang X, Zhang W, Li H, Zhao W, Sun J, Yang M (2020). Melatonin suppresses ferroptosis induced by high glucose via activation of the Nrf2/HO-1 signaling pathway in type 2 diabetic osteoporosis. Oxid Med Cell Longev.

[42] Li J, Lu K, Sun F, Tan S, Zhang X, Sheng W, Hao W (2021). Panaxydol attenuates ferroptosis against LPS-induced acute lung injury in mice by Keap1-Nrf2/HO-1 pathway. J Transl Med.

[43] Ayer A, Zarjou A, Agarwal A, Stocker R (2016). Heme oxygenases in cardiovascular health and disease. Physiol Rev.

