# Supplementary material for: Inhibition of HMOX1 alleviates diabetic cardiomyopathy by targeting ferroptosis: Inhibition of HMOX1 alleviates diabetic cardiomyopathy
Source: Acta Biochim Biophys Sin (Shanghai). 2025 Apr 16;57(9):1420–32. doi: 10.3724/abbs.2024232 (PMC12536467; doi:10.3724/abbs.2024232)
Supplement: 24696Supplementary_data [file 24696Supplementary_data.docx]

**Supplementary Table S1.** **Sequences of primers used in RT-qPCR**

| Gene | Species | Primer sequence (5′→3′) | |
| --- | --- | --- | --- |
| *PTGS2* | *Mus* | Forward | TGAGTACCGCAAACGCTTCT |
|  |  | Reverse | CAGCCATTTCCTTCTCTCCTGT |
| *ACSL4* | *Mus* | Forward | CTTCCTCTTAAGGCCGGGAC |
|  |  | Reverse | TCTCTTTGCCATAGCGTTTTTAGAT |
| *HMOX1* | *Mus* | Forward | CAGAGCCGTCTCGAGCATAG |
|  |  | Reverse | CAAATCCTGGGGCATGCTGT |
| *β-actin* | *Mus* | Forward | ACTATTGGCAACGAGCGGTTCC |
|  |  | Reverse | GCACTGTGTTGGCATAGAGGTCTT |
| *PTGS2* | *Rattus* | Forward | CTCAGCCATGCAGCAAATCC |
|  |  | Reverse | GGGTGGGCTTCAGCAGTAAT |
| *ACSL4* | *Rattus* | Forward | GCAGCACCTTCGATCCCA |
|  |  | Reverse | GCGTGACAGAGCGATATGGA |
| *HMOX1* | *Rattus* | Forward | TGCACATCCGTGCAGAGAAT |
|  |  | Reverse | AGGAGGCCATCACCAGCTTA |
| *β-actin* | *Rattus* | Forward | CCCGCGAGTACAACCTTCTTG |
|  |  | Reverse | GTCATCCATGGCGAACTGGTG |

**
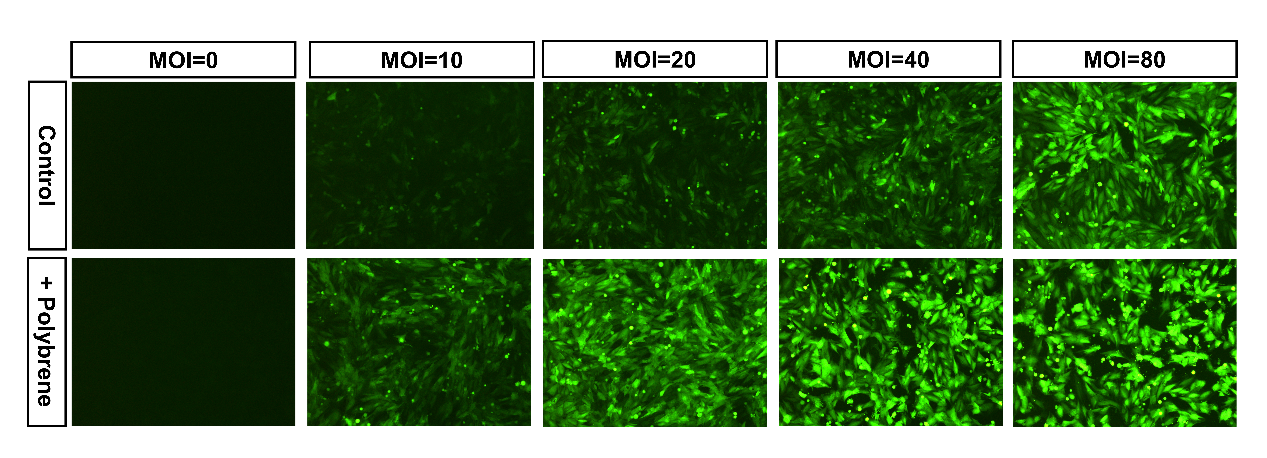
**

**Supplementary Figure S1. Lentivirus transfection at different MOI values with or without polybrene**


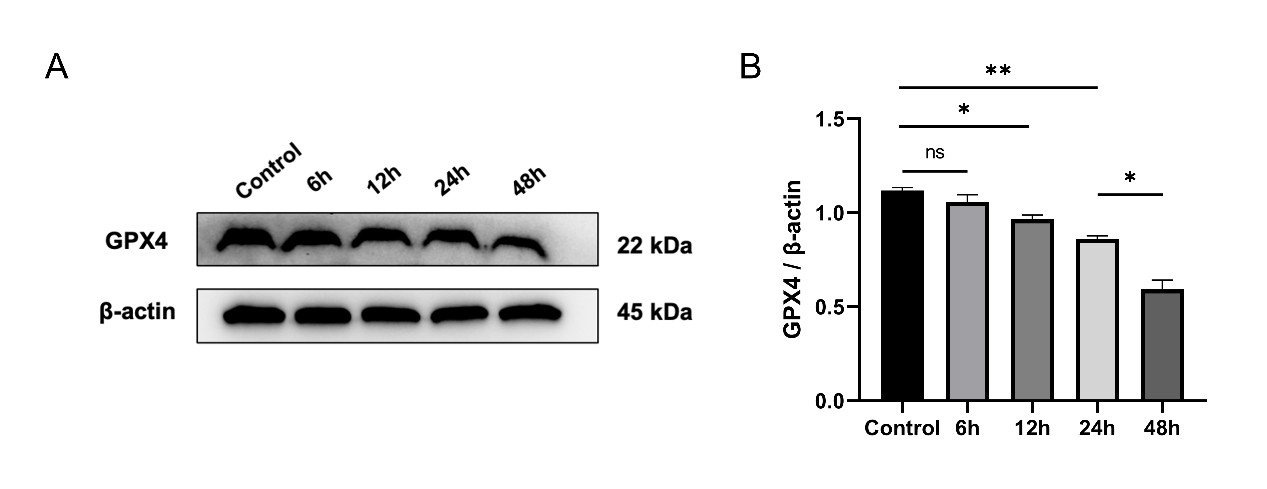


**Supplementary Figure S2. GPX4 expression changes in H9C2 cells treated with GP for different time** (A) GPX4 protein expression in H9C2 cells treated with GP for 6 h, 12 h, 24 h, and 48 h determined by western blot analysis. (B) Quantitative analysis of GPX4 expression in 5 groups. ^ns^*P*>0.05, **P*<0.05, ***P*<0.01.
